# Obesity paradox and lung cancer, metformin-based therapeutic opportunity?

**DOI:** 10.18632/oncotarget.28432

**Published:** 2023-07-01

**Authors:** Pedro Barrios-Bernal, Norma Hernández-Pedro, Luis Lara-Mejía, Oscar Arrieta

**Keywords:** metformin, tyrosine-kinase inhibitors, BMI, EGFR, obesity paradox

Obesity is a complex multifactorial disease with detrimental effects on health. This disease induces a pro-inflammatory state, innate and adaptative immune system dysfunction, and immune exhaustion, which in conjunction promote cancer growth. Although obesity and type 2 diabetes mellitus (T2DM) have been associated with lung cancer (LC) development, several confounding factors, such as chronic inflammation, high insulin levels, microbiome, as well as the oncogenic potential of growth and sexual hormones, have introduced uncertainty and avoid the fully recognition of this relationship [1, 2]. Thus, therapies that can bring potential therapeutic effects to both comorbidities are being tested globally and their effect on cancer cells.

In detail, this biguanide has been related to several metabolic mechanisms, signaling pathways, and glucose uptake in concomitant use with standard anticancer therapies, such as radiotherapy (RT), chemotherapy, tyrosine-kinase inhibitors (TKIs), and immunotherapy [3]. Moreover, metformin acts over cell metabolism inhibiting complex I of the electron transport chain, and cell metabolic stress through AMPK pathway activation, which subsequently inhibits the mammalian target of rapamycin (mTOR) and its downstream effectors. Through this mechanism, metformin reduce protein synthesis, anabolic processes and proliferation [4]. In cancer cells, metformin has been shown to modify tumor metastatic properties by cell migration inhibition and suppressing epithelial-mesenchymal transition (EMT). Similarly, positive immunogenic properties have been associated with metformin through the activation of cytotoxic T- cells and enhancement of oxidative metabolism, which promotes an antitumor immune response [3, 5].

Metformin in combination with antineoplastic therapies has been tested in lung cancer models, promoting apoptosis, autophagy, and cell cycle arrest [3]. Therefore, lately has raised a particular interest in emerging metabolic-modifying therapies such as metformin, seeking to defeat cancer-promoting conditions caused by obesity. In one clinical study, patients with non-small cell lung cancer and early-stage disease who underwent lobectomy showed a higher disease-specific survival (DSS) and overall survival (OS) in patients with a body mass index (BMI)≥24 and metformin intake. Conversely, no survival benefits have been reported in patients with a BMI<24, which contributes to the formulation of the “obesity paradox” [6]. Yendamuri and colleagues also underscore about the positive interaction of obesity and metformin, in which those patients with a BMI>30 kg/m^2^ reported even a more prolonged OS and DSS. Of interest, in a separate cohort of advanced-stage LC tumors, downregulation of immune checkpoint gene expression (PD-L1, CTLA4, LAG3) was withheld in metformin users and high BMI, reinforcing the potential implication of leptin as a mediator of obesity associated immune disfunction and its restoration with metformin [7].

Likewise, our group conducted one phase 2 trial that assessed the benefits of adding metformin to TKI therapy in advanced EGFR mutant NSCLC. In the post hoc analysis, we found a positive interaction between BMI and the benefits of metformin administration plus the investigator-chosen TKI. This analysis demonstrated a longer progression-free survival (PFS) and OS in those patients who received metformin plus EGFR-TKI compared with the EGFR-TKI administration only (15.83 vs. 8.34 months; HR 0.47, *p* = 0.003) and (31.4 vs. 18.0; HR 0.55, *p* = 0.04). Our data in advanced EGFR mutant NSCLC reinforced at that moment what Yendamuri et al. found in earlier stages; a selective effect in the obese population, and a lack of benefit in patients with a BMI<24. These findings suggest a strongly sensitization by the addition of metformin in obese population, suggesting that biochemical and molecular differences influence the treatment response [8].

Obesity paradox and different responses to metformin treatment may be attributable to diverse factors; among which it takes special importance the capacity of metformin to reduce cellular senescence. It has been explored this effect through the inhibition of oxidative stress and reduction of mitochondrial dysfunction in adipocytes promoting stimulation and aging inhibition. Similarly, metformin can offer aging protection in non-transformed cells when radiotherapy is administered. It is known that obesity is a recurrent factor in senescent immune systems, in which metformin can attenuate the aging effect through the prevention of T-cell exhaustion and promoting T-cell activation. The anti-aging effect of metformin recently have gained interest, due to the molecular and biochemical mechanisms of cell aging being related to several diseases including some cancer types.

## CONCLUSION

Altogether, there is a strong relationship between high BMI and increase survival in different LC stages and in combination with some anticancer therapies. Metformin has been shown to be a metabolism modifier that may adapt signaling pathways and immune sensitivity in tumor microenvironment. As most of the mechanisms involved in this phenomenon are currently unexplored, further investigation is needed to determine whether any of these proposed mechanisms has any clinically meaningful activity in the treatment of obese patients with LC. Until then, we propose that pharmacodynamics, pharmacokinetics, metabolic parameters, tumor biology, biochemical and molecular modifications may be related to “obesity paradox” and must be taken into account to choose the most appropriate treatment.
